# MicroRNA Therapeutics in Cancer: Current Advances and Challenges

**DOI:** 10.3390/cancers13112680

**Published:** 2021-05-29

**Authors:** Soha Reda El Sayed, Justine Cristante, Laurent Guyon, Josiane Denis, Olivier Chabre, Nadia Cherradi

**Affiliations:** 1 University Grenoble Alpes, INSERM, CEA, Interdisciplinary Research Institute of Grenoble (IRIG), Biology and Biotechnologies for Health UMR_1292, F-38000 Grenoble, France; soha.redaelsayed@cea.fr (S.R.E.S.); jcristante@chu-grenoble.fr (J.C.); laurent.guyon@cea.fr (L.G.); josiane.denis@cea.fr (J.D.); OlivierChabre@chu-grenoble.fr (O.C.); 2 Centre Hospitalier Universitaire Grenoble Alpes, Service d’Endocrinologie, F-38000 Grenoble, France

**Keywords:** cancer, preclinical research, multi-target therapy, microRNA delivery, nanotechnology, nanoparticles, nanomedicine platforms

## Abstract

**Simple Summary:**

Cancer is a complex disease associated with deregulation of numerous genes. In addition, redundant cellular pathways limit efficiency of monotarget drugs in cancer therapy. MicroRNAs are a class of gene expression regulators, which often function by targeting multiple genes. This feature makes them a double-edged sword (a) as attractive targets for anti-tumor therapy and concomitantly (b) as risky targets due to their potential side effects on healthy tissues. As for conventional antitumor drugs, nanocarriers have been developed to circumvent the problems associated with miRNA delivery to tumors. In this review, we highlight studies that have established the pre-clinical proof-of concept of miRNAs as relevant therapeutic targets in oncology. Particular attention was brought to new strategies based on nanovectorization of miRNAs as well as to the perspectives for their applications.

**Abstract:**

The discovery of microRNAs (miRNAs) in 1993 has challenged the dogma of gene expression regulation. MiRNAs affect most of cellular processes from metabolism, through cell proliferation and differentiation, to cell death. In cancer, deregulated miRNA expression leads to tumor development and progression by promoting acquisition of cancer hallmark traits. The multi-target action of miRNAs, which enable regulation of entire signaling networks, makes them attractive tools for the development of anti-cancer therapies. Hence, supplementing downregulated miRNA by synthetic oligonucleotides or silencing overexpressed miRNAs through artificial antagonists became a common strategy in cancer research. However, the ultimate success of miRNA therapeutics will depend on solving pharmacokinetic and targeted delivery issues. The development of a number of nanocarrier-based platforms holds significant promises to enhance the cell specific controlled delivery and safety profile of miRNA-based therapies. In this review, we provide among the most comprehensive assessments to date of promising nanomedicine platforms that have been tested preclinically, pertaining to the treatment of selected solid tumors including lung, liver, breast, and glioblastoma tumors as well as endocrine malignancies. The future challenges and potential applications in clinical oncology are discussed.

## 1. Introduction

MicroRNAs (miRNAs) are highly conserved small non-coding RNAs, which regulate gene expression through imperfect base pairing to the 3′-untranslated region (3′-UTR) of target mRNA. For the most part, miRNA binding through partial complementarity to the target transcript leads to its degradation or repression of its translation [1]. MiRNAs have a particular biogenesis that makes their expression both spatially and temporarily controlled [2]: miRNA genes are transcribed into hairpin-containing primary transcripts (pri-miRNA). Pri-miRNAs are cleaved by the Double-Stranded RNA-Specific Endoribonuclease DROSHA (RNase III) and its cofactor DiGeorge syndrome Critical Region 8 (DGCR8) into short hairpin pre-miRNAs in the nucleus. Pre-miRNAs are then transported into the cytoplasm by an Exportin-5 RanGTP complex to undergo further processing into approximately 22 nucleotides-double-stranded mature miRNAs by the Dicer RNase III/TRBP (HIV-1 transactivating response (TAR) RNA-binding protein) complex. The resulting small RNA duplex is then assembled into AGO (Argonaute) protein within the RNA-Induced Silencing Complex (RISC) where the guide strand is selected to exert its effect on the target transcript [3].

Given their small size of ~22 nucleotides, miRNAs can regulate various genes, in a developmental and tissue-specific manner [4]. To date, about 1917 human precursors and 2654 mature miRNAs have been described in miRBase (http://www.mirbase.org/; accessed on 22 October 2018), some of which have been implicated in human pathologies. Their involvement in cancer was first demonstrated in 2002, when miR-15 and miR-16-1 were found to be downregulated in Chronic Lymphocytic Leukemia [5]. Since then, high throughput molecular profiling allowed detection of aberrant miRNA expression in various tumors as compared to healthy tissue [6]. These cancer-associated miRNA signatures result from alterations of several mechanisms including structural genetic alterations (chromosomal deletions/amplifications and mutations), defects in the miRNA biogenesis machinery [7], and epigenetic changes such as altered DNA methylation [8]. Tumor hypoxia is also a key regulator of miRNA expression. Notably, Hypoxia Inducible Factor-1α (HIF-1α) downregulates miR-34a, thus promoting epithelial to mesenchymal transition by targeting the Notch signaling pathway in epithelial cells [9]. Further genetic studies indicated that the specific localization of more than 50% of miRNA genes in fragile genomic regions favors their imbalanced expression, thus their involvement in tumorigenesis. In general, miRNAs embedded in cancer-deleted loci (such as the miR-15a-miR-16-1 cluster at 13q14) act as tumor suppressors, whereas miRNAs located in cancer-amplified genomic regions (such as the miR-17-92 cluster) function as oncogenes [10].

In addition to their major involvement in tumorigenesis and metastasis, miRNAs have been linked to drug resistance, the principal limiting factor to achieving cures in patients with cancer [11,12]. Indeed, alterations in miRNA expression profiles lead to anticancer drug resistance by abnormally regulating the expression of genes involved in multi-drug-resistance (MDR) mechanisms, such as ATP-binding cassette (ABC) transporter genes, apoptosis- and autophagy-related genes, and drug metabolism-related genes [13]. For example, over-expression of miR-223 or miR-298 in doxorubicin (DOX)-resistant hepatocellular carcinoma (HCC) cells or in breast cancer cells, respectively, increased their sensitivity to DOX through ABCB1 (ABC Subfamily B Member 1) downregulation [14,15]. In chronic myeloid leukemia, miR-212 inhibition resulted in ABCG2 (ABC Subfamily G Member 2) upregulation and increased ABCG2-dependent efflux of Imatinib [16]. Knockdown of miRNA-182 and miRNA-205 improve the sensitivity of non-small-cell lung cancer (NSCLC) to cisplatin, and enhanced apoptosis through upregulation of the pro-apoptotic proteins phosphatase and tensin homolog (PTEN) and programmed cell death-4 (PDCD4), respectively [17,18]. Autophagy is activated in cancer cells during chemotherapy and often contributes to drug resistance [19]. Zou et al. found that ectopic expression of miRNA-30a significantly reduced beclin 1 and cisplatin-induced autophagy while significantly increasing HCC and breast cancer cell apoptosis [20]. MiRNAs also regulate drug-metabolizing enzymes such as the cytochrome P450 (CYP) superfamily, which catalyzes the metabolism of most drugs. As observed for ABC transporters, the expression level of drug-metabolizing enzymes is frequently higher in various types of cancers compared with normal tissues. MiR-27b and miR-892a were found to respectively target and downregulate CYP1B1 and CYP1A1 expression in breast cancer [21,22] and to impair the benzo(a)pyrene-mediated decrease in cancer cell viability [22]. All these findings reinforce the idea that subsets of miRNAs may have clinical relevance as therapeutics agents.

Besides their validation as powerful tools for diagnosis, inhibition of miRNA activity and/or enhancement of miRNA function (miRNA replacement) strategies led to promising results in terms of antitumor effects in preclinical models [23,24,25,26]. It is worth mentioning that the development of miRNA-based therapies continues to benefit from the major advances made in siRNA/RNA therapeutics. As components of the RNA interference (RNAi) process, both miRNA and siRNA are able to knockdown oncogenic genes by targeting mRNA expression. MiRNA and siRNA have similar physicochemical properties (double-stranded RNA with 21–23 nucleotides) and use the same intracellular machinery to be active (function of the RNA-induced Silencing Complex). Therefore, it is conceivable that similar technologies can be applied to both types of RNA for therapeutic purposes. However, the origin and mechanisms of action of miRNA and siRNA differ: miRNA are encoded by the cell genome and regulate endogenous genes while siRNA function after exogenous delivery; miRNA mostly use 7–8 nucleotides from their 5′-end to identify target mRNA sequence and to induce mRNA degradation or inhibition of translation. Consequently, a single miRNA is able to bind and target more than one mRNA, thus allowing multi-target action on several genes, which often work together as a network within the same pathway [27]. This property is attractive for the treatment of multifactorial diseases such as cancer but can also lead to potential off-target effects. In contrast, siRNA use their full length to recognize their target sequence and mediate cleavage of the target mRNA, thus permitting target specificity and the ability to inhibit the expression of a mutant oncogenic protein without affecting the wild type. Nevertheless, siRNAs can in turn cause unintended gene silencing due to miRNA-like effects when their 5′-end of the guide strand is complementary to the 3′-UTR of the mRNA.

**Figure 1:**
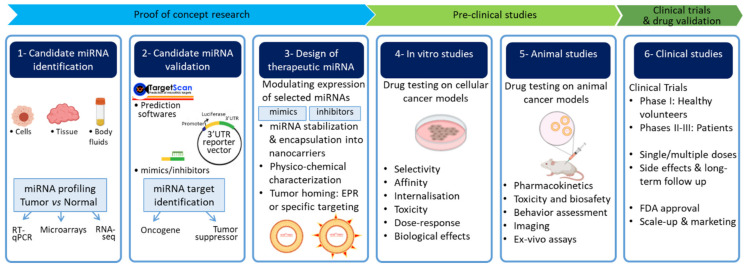
Translating miRNA biology from bench to bedside in cancer. As for the classical drug discovery workflow, development of miRNA therapeutics consists of 3 main levels: proof of concept research, preclinical studies, and clinical trials. (**1**) Identification of candidate miRNAs for therapy. MiRNA expression is quantified in tissue, cells, or body fluids of healthy and tumor specimens (RT-qPCR: Reverse Transcription-quantitative PCR; RNA-Seq: RNA sequencing). (**2**) Potential targets of differentially expressed miRNAs can be identified using target prediction softwares and validated in reporter gene assays vectors using target transcript 3′-UTR cloned downstream of luciferase reporter and miRNA mimics/inhibitors. (**3**) Design of therapeutic miRNA requires stabilization and encapsulation of miRNAs in well characterized carriers. (**4**) Evaluation of the effects of miRNA-loaded nanocarriers on several biological processes in cancer cell models is a pre-requisite for the development of therapeutic protocols in vivo. (**5**) Therapeutic miRNA candidates are tested in animal cancer models alongside animal behavior and recovery before the evaluation of the antitumor effects. (**6**) Initiation of clinical trials requires a careful assessment of efficacy and toxicity in pre-clinical studies. Doses and side effects are particularly monitored for FDA approval and treatment scale-up.

A series of stringent criteria must be met before bringing miRNAs from bench to bedside (Figure 1). These include safe delivery, limitation of off-target effects which are inherent to miRNA mechanisms of action, and reduction of toxicity and immune responses. In this review, we summarize the emergence of miRNA-based therapy as a strategy to treat cancer by specifically targeting signaling pathways leading to the disease. We cover the approaches implemented for the delivery of miRNA mimics or anti-miRNAs (antimiRs) with an emphasis on nanotechnology-based formulations for the treatment of major cancer types and rare endocrine tumors in preclinical models. The challenges that persist for translating laboratory breakthroughs to the clinic are discussed.

## 2. Main Approaches for Therapeutic Targeting of miRNAs

MiRNA expression patterns can be modulated to abolish or restore miRNA biological function. To inhibit oncogenes or restore tumor suppressors, one anti-cancer strategy consists of silencing the overexpressed oncomiRs or replacing the downregulated tumor suppressor miRNAs [28]. There are three approaches to achieve miRNA loss of function: miRNA sponges, antisense oligonucleotides (antagomiRs, antimiRs), and genetic knockouts based on the application of Clustered Regularly Interspaced Short Palindromic Repeats/CRISPR-associated protein 9 (CRISPR/Cas9) genome-editing technologies [29,30]. Synthetic miRNA sponge vectors express transcripts with miRNA binding sites that mimic those found in natural mRNAs and complementary to the targeted miRNA [31]. This system sequesters endogenous intracellular miRNAs, thus preventing their binding availability for the target mRNAs [32]. By transducing a retroviral miRNA sponge to inhibit miR-9, Ma et al. demonstrated that metastasis was significantly reduced in a syngeneic mouse model of breast cancer [33]. High affinity-inhibition is also feasible via chemically modified oligonucleotides such as locked nucleic acids (LNA). As a part of the cell endogenous DNA repair machinery, the CRISPR/Cas9 system has been reported recently as a potent genetic engineering tool for miRNA-based therapeutic intervention. Yoshino and colleagues targeted miR-210-3p and miR-210-5p using the CRISPR/Cas9 system in renal cell carcinoma cell lines and demonstrated that deletion of miR-210-3p increased tumorigenesis, both in vitro and in vivo [34]. Another growing field in miRNA therapeutics is miRNA replacement therapy which aims at restoring miRNAs, which are downregulated or deleted in cancer cells [35]. With the recurrence of downregulated tumor suppressor miRNAs in human malignancies, mainly miR-34 and let-7, administration of miRNA mimics can re-establish miRNA levels to their basal non-pathological states. Indeed, a decrease of let-7 promotes expression of a number of oncogenic factors, including RAS, Myc, cyclins, and cyclin-dependent kinases [36]. In cultured lung cancer cells as well as in pre-clinical models of lung cancer, re-introduction of let-7 mimics impedes cell proliferation and reduces growth of lung tumors [37]. MiR-34a is markedly under-expressed in most human cancer types. Re-expression of miR-34a induces growth arrest and apoptosis, by silencing pro-proliferative and anti-apoptotic genes [38].

## 3. Delivery Platforms for miRNA Therapeutics

Improvement of miRNA mimics or antimiRs stability and development of safe and efficient delivery systems are critical steps to bring miRNA therapies from bench to bedside. Indeed, synthetic miRNA mimics or antimiR oligonucleotides have short half-life and are immediately degraded in biological fluids by nucleases [39]. To overcome this hurdle, several strategies have been devised, including chemical modifications such as phosphodiester and phosphorothioate internucleotide linkages, addition of a 2′-*O*-methyl group or synthesis of locked nucleic acids in which the ribose ring is constrained by a methylene linkage between the 2-oxygen and the 4-carbon. In addition to chemical modifications, entrapment of therapeutic miRNAs within functionalized nanoparticles allowed further improvement in their protection from degradation, decreased the immune response and enhanced the circulation time. Finally, conjugation of nanoparticles with targeting ligands such as proteins, peptides, and antibodies improved cellular uptake and specific targeting of the tumor site.

Several viral and non-viral miRNA delivery systems have been used successfully in vitro and in vivo. Nevertheless, whether based on chemically modified oligonucleotides, miRNA sponges or miRNA mimics, developing therapeutic approaches still present clearance, accessibility, tissue-specific targeting and safety issues [40]. The exponential growth in nanotechnology research is expected to help to overcome these barriers: oligonucleotides can be encapsulated into complex nanoparticles (NPs) capable of efficient and targeted drug delivery. Besides improved endosomal escape, these nanocarriers achieve tumor-selective accumulation through the Enhanced Permeability and Retention (EPR) effect, a central paradigm in cancer nanomedicine [41]. This passive targeting mechanism results from the extravasation of long-circulating nanoparticles (diameter < 100 nm) through the leaky tumor microvasculature into the tumor interstitium. Subsequent nanoparticle cellular uptake and intracellular fate are strongly influenced by their size, shape and surface properties [42].

**Figure 2:**
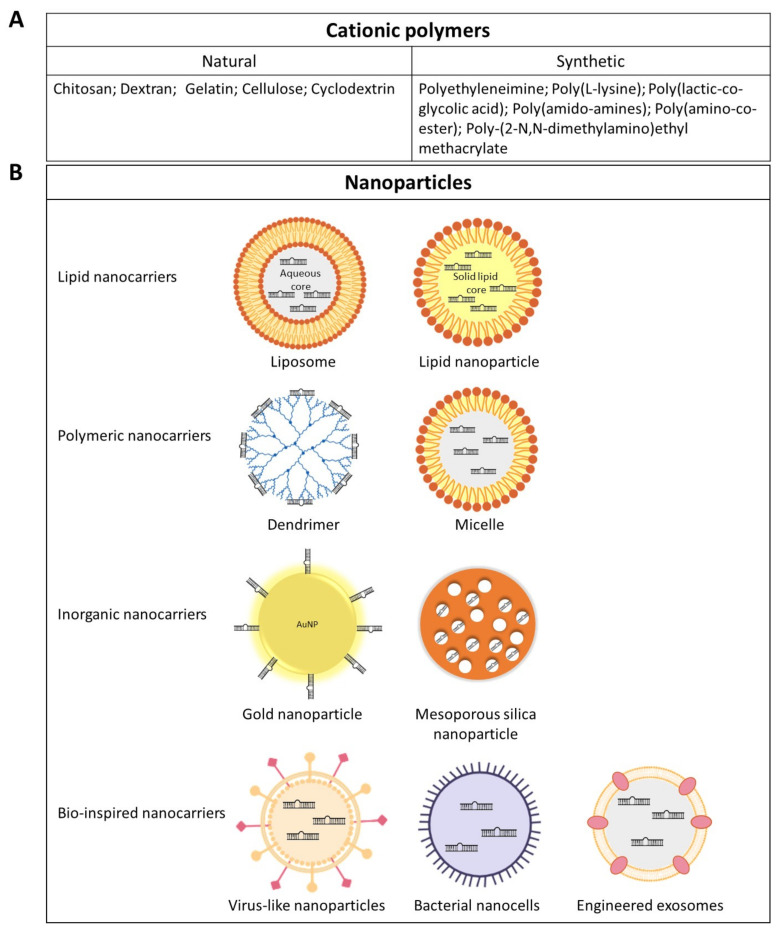
Schematic representation of commonly used and emerging nanoplatforms for miRNA delivery. (**A**) Natural and synthetic polymers can form electrostatic complexes with nucleic acids such as miRNAs. (**B**) Nanoparticle-based platforms are characterized by tunable size, shape, and surface characteristics, which enable them to have compatibility with different administration routes. Specific recognition molecules such as antibodies or peptides can be grafted to target tissues more specifically. Tumor-derived exosomes are being increasingly explored as delivery systems in cancer research since their identification as drivers of organotropic metastatic spread. However, their complex composition and still non-established biological functions led to the development of Exosome-Mimetic Nanosystems that recapitulate natural exosomes structure with a controlled composition.

Genetically modified viral vectors, including retroviruses, lentiviruses, adenoviruses and adeno-associated viruses (AAVs) have long been used for gene therapy and also designed to deliver transgenes encoding miRNA mimics or antagonists [43]. Virus-like nanoparticles (VLNPs) are noninfectious protein shells or capsids, composed of virus-derived structural proteins and devoid of the pathogenic elements of the viral genome. VLNPs can be produced from infections of host cells or by recombinant protein expression and self-assembly. The advantage of viral vectors is to provide high infection efficiency and persistent expression of the transgene. For example, systemic lentiviral delivery of miR-15a/16 in a mouse model of Chronic Lymphocytic Leukemia restored the expression of miR-15a/16, reduced malignancy with decreased proliferation and increased apoptosis of malignant lymphoid cells [44]. However, lentiviruses and retroviruses can integrate their own reverse transcribed DNA into the host genome, which may lead to insertional mutagenesis and activation of oncogenic pathways. Thus, non-integrating adenoviruses and AAVs have been used as alternative miRNA carriers as they keep their own genomes in episomal form. For example, systemic delivery of miR-26a carried by AAVs showed cell cycle arrest and apoptosis induction in hepatocellular carcinoma cells and tumor growth inhibition [45]. Although the viral vectors used are replication-deficient, some problems such as toxicity, immunogenicity, and manufacturing complexity shifted the research in nanomedicine towards non-viral carriers. Thus, polymeric non-viral vectors, which have been favored due to their low immunogenicity, ease of production, controlled composition, and chemical flexibility, have represented an attractive alternative to viral vectors (Figure 2).

Various types of natural and synthetic polymers have been used in miRNA-based therapies. Interest in synthetic cationic polymers resulted from their potential to form polyelectrolyte complexes with nucleic acids. Polyethyleneimine (PEI), an organic macromolecule with a high cationic-charge-density potential, is the most commonly used polymeric gene delivery system. The overall positive charge of PEI makes it convenient for condensing large negatively charged molecules such as nucleic acids, resulting in the formation of polyplexes through electrostatic complexation. Following endocytosis, PEI undergoes protonation of its amine groups within endosomes and thereby exerts a proton-sponge effect. Proton accumulation triggers cytosolic water towards the endosomes, leading therefore to osmotic swelling, endosome bursting, and PEI polyplexe release into the cytosol [46]. Systemic or local application of PEI/miR-145 complexes into a mouse model of colon carcinoma significantly reduced tumor proliferation and increased apoptosis, with concomitant repression of c-Myc and ERK5 [47]. Natural cationic polymers including chitosan and dextran (polysaccharides) were also successfully tested for miRNA delivery to treat multiple myeloma and osteosarcoma in preclinical models [48,49]. Chitosan has a strong binding affinity for nucleic acids at low pH as its protonated amine groups rapidly interact with negatively charged molecules such as miRNAs. A major drawback of chitosan nanoparticles is that these interactions are almost irreversible thus preventing efficient drug release. Lipid chains or negatively charged polymers have been combined with chitosan to improve nucleic acid delivery [50].

Other studies investigated the potential of inorganic materials such as gold (Au) or silica NPs in miRNA-based therapy. Inorganic NPs feature several advantages, including tunable size, surface properties, and multifunctional capabilities. Multiple strategies have been used for the functionalization of Au-NPs to increase their bonding with biological molecules and facilitate the intracellular payload release. Gold nanoparticles (Au-NPs) can be functionalized with thiol groups to increase their bonding with miRNA [51]. This approach has been reported by Ekin et al. to successfully convey miR-145 to prostate PC3 and breast MCF-7 cell lines [52]. An additional polyethylene glycol (PEG) layer was shown to stabilize Au-NPs nanoformulations by limiting their aggregation and miRNA degradation [53]. Moreover, Au-NPs binding with the target site can be addressed by decorating their surface with target specific ligands. Even though AU-NPs have received a lot of interest over the past few years, more investigations related to biocompatibility, cytotoxicity, retention, and clearance time are needed to conceive conjugated Au-NPs with minimal side effects [54,55]. Mesoporous silica nanoparticles (MSN) are a special group of inorganic NPs that have porosities at the nanoscale. They provide a high surface area, thermal stability, and easy surface modification, with biocompatible and non-toxic properties. Their large and active surface allows the attachment of different functional groups for targeted drug delivery. Among the many strategies that are used to functionalize MSNs, chemical modifications within the pores to increase the retention time of loaded molecules, coating with PEG for stabilization and attachment of targeting ligands to target specific cell receptors have been extensively investigated. Tivnan et al. exploited the high expression level of the tumor-associated antigen disialoganglioside (GD2) in neuroblastoma to develop GD2-targeting MSN for the delivery of miR-34 into neuroblastoma murine models [56]. However, the synthesis of functionalized MSN requires multiple steps with complex chemical reactions that limited their fabrication at industrial scale.

Lipid-based nanoparticles (LNPs) are widely used due to their efficient cellular uptake through the cell membrane. Different types of nanoformulations, such as liposomes and solid lipid nanoparticles (SLNs) prove to be less toxic than other delivery systems such as polymer nanoparticles, owing to their biocompatibility and biodegradability. MiRNA-loaded LNPs are usually a cocktail of cationic lipids (*N*-[1-(2,3-dioleyloxy)propyl]-*N*,*N*,*N*-trimethylammonium chloride (DOTMA) or 1,2-dioleoyl-3-trimethylammonium-propane (DOTAP)), neutral lipids and PEG, which shield miRNAs either in their aqueous core or by forming a stable complex via electrostatic interactions with the negatively charged phosphate groups in miRNA molecules [57]. Helper lipids, i.e., neutral lipids like cholesterol and dioleoylphosphatidyl ethanolamine (DOPE), can be incorporated in LNPs in order to reduce the charge-driven toxicity and to enhance delivery efficiency [58,59]. LNPs increased the therapeutic index of many drugs and offered improved drug targeting and controlled release. As for the other drug delivery nanosystems, targeted liposomal formulations have been developed by coating liposomes with specific ligands, which bind to cancer-associated antigens. For example, taking advantage of the selective internalization of GAH antibodies by gastric cancer cells, Hosokawa et al. showed that doxorubicin exerted better anti-tumor activity when vectorized in PEG-GAH liposomes than in non-coated liposomes [60,61]. In the context of miRNA-based therapy, miR-135a-loaded cationic immunoliposomes coated with anti-EGFR (Epidermal Growth Factor Receptor) antibodies (Anti-EGFR-CIL-miR-135a) were shown to inhibit gallbladder carcinoma invasion (GBC) and metastasis, and to promote apoptosis. The GBC tumor growth rate was 60% lower in xenograft-bearing mice treated with Anti-EGFR-CIL-miR-135a as compared to controls [62]. PD-L1 monoclonal antibody-conjugated miR-130a/oxaliplatin-loaded immunoliposomes (PD-miOXNP) showed a high efficacy in HGC27 gastric cancer tumors with reduced Ki67+ cells and increased TUNEL+ cells [63]. SLNs offer additional advantages over polymeric NPs and liposomes. Indeed, incorporation of both hydrophilic and hydrophobic drugs is achievable along with controlled release of the drug for up to several weeks [64]. Moreover, the lipids used in the preparation of SLNs are biodegradable and safe. SLN formulations are also characterized by a high stability and loading capacity as compared to their lipid counterparts. The main components of SLN formulation are solid lipids, surfactants, and water. Solid lipids include fatty acids, triglycerides, steroids, and waxes. Cationic lipids facilitate interaction with the cell membrane, improving transfection efficiency. Combining miRNA with chemotherapeutic drugs using SLNs was shown to be a powerful anticancer strategy. Shi et al. demonstrated that co-incorporation of miR-34a and paclitaxel (PTX) in SLNs (miSLNs-34a/PTX) increases the uptake of these nanoparticles by B16F10-CD44+ melanoma cells and induces more cell death than single drug-loaded nanoparticles [65]. MiR-34a and PTX exerted a synergistic induction of melanoma cell death. In another study, cationic SLNs were successfully used to deliver anti-miR-21 oligonucleotide and Pemetrexed for glioblastoma therapy in vitro [66]. Micelles are nanoparticles that are formed from the self-assembling of amphiphilic molecules in an aqueous environment. Reported advantages of micelles include simple preparation, low toxicity and good tissue penetration properties [67]. However, similar to liposomes, they are prone to dilution following intravenous administration. Modifications of micelles at their core and their shell can improve encapsulation efficiency and in vivo stability. Mittal et al. designed gemcitabine-conjugated cationic micelles for the co-delivery of gemcitabine and miRNA-205 in pancreatic cancer [68]. Combination formulations efficiently reversed chemoresistance, invasion and migration in gemcitabine-resistant pancreatic cancer cells in vitro, and showed significant growth inhibition in vivo.

A powerful delivery vehicle based on bacterially derived nanocells, called EDV™ (EnGeneIC Dream Vectors) has been developed by EnGeneIC Ltd. (Sydney, Australia) [69]. Bacterial nanocells are achromosomal nanoparticles produced by inactivation of the genes that control normal bacterial cell division. They can package a range of anticancer chemotherapeutic drugs [70]. Targeted delivery was achieved by using bispecific antibodies, which are capable of binding the EDVs with one arm and the tumor antigen with the other arm. In addition, the bacterial cell wall of the nanocells stimulates key components of the immune system, which are then activated to kill cancer cells. The EDVs proved to be safe and well tolerated despite high and repeated doses in different animal models [70,71]. This system has been used to deliver miR-16 to mesothelioma in vivo [72] as well as to mesothelioma patients (MesomiR-1 clinical trial NCT02369198). Another elegant strategy for miRNA delivery was inspired by natural exosomes, which shield and convey miRNAs into the tumor niche. Nevertheless, besides the validation of exosomes as biocompatible molecular carriers, their clinical translation is still hampered by their complex composition and poor harvesting yields [73]. To overcome these issues, Vazquez-Rios et al. took advantage of the existing liposome technology to develop Exosome-Mimetic Nanosystems (EMNs) [74]. These nanostructures reproduce cell-derived exosomes structure, physicochemical properties, and loading capacities.

## 4. Application of miRNA-Based Therapeutics in Selected Cancers

In their recent review, Bonneau et al. reported the clinical advances for miRNA therapeutics in several human diseases, including cancer [75]. In the following sections, we will describe the preclinical advances in anti-cancer strategies using miRNA-based therapy for selected common and rare solid tumors. Table 1 provides several examples of in vitro and in vivo studies using different delivery systems and administration routes to replace or inhibit miRNAs in cancer cells.

### 4.1. Lung Cancer

Lung cancer is the most frequent cause of cancer-related deaths worldwide with 5-year survival rates ranging from 4–17% depending on stage and populations. Liposomes have been reported to be particularly convenient for drug delivery to the lungs, since they can be prepared from lung endogenous surfactants. This makes them relevant carriers for miRNA-targeting molecules to this organ. To date, miR-34a is the most well documented tumor suppressor miRNA, capable of cell cycle arrest and induction of apoptosis. Its downregulation is reported in various solid tumors including lung cancer, suggesting that replacement therapy might be effective for retrieving its physiological levels [76]. Wiggins et al. [77] showed that systemic delivery of synthetic miR-34a in liposomal formulation could indeed inhibit tumor growth in NSCLC-bearing mice. No immunogenicity or toxicity were observed. These results were in agreement with prior in vitro experiments on genetic variants of NSCLC cell lines, showing that transfection of miR-34a reduced cell growth and colony formation in a p53 dependent manner [77]. In the same line, Kasinski et al. suggested a combinatorial approach to co-deliver the tumor suppressors miR-34 and let-7b using NOV340 liposomes in NSCLC. This strategy reduced tumor burden and induced a 40%-increase in survival rate of Kras^G12D/+^/Trp53^flx/flx^ mutant mice [78]. Systemic delivery of miR-200c loaded-NOV340 liposomes has been shown to enhance radiosensitivity in lung cancer by increasing the oxidative stress response and by inhibiting repair of radiation-induced DNA double-strand breaks [79]. Thus, rendering treatment-resistant lung cancer sensitive to radiotherapy through lipid nanoparticle-mediated miRNA replacement appeared as a promising approach. On the clinical side, miR-34 mimics, encapsulated in NOV340 liposomes (MRX34), were the first miRNA-based therapy approach that entered phase I clinical testing in 2013 for several solid and hematological malignancies (NCT01829971, Mirna Therapeutics) [80]. Unfortunately, this study was halted in 2016 following multiple immune-related severe adverse events observed in the patients [81].

Another approach demonstrated that DOTMA-based cationic lipoplexes (LPs) successfully conveyed miR-29b (LP-miR-29b) to both NSCLC A549 cells in culture and NSCLC xenograft mouse model [82]. After several injections of miR-29b-loaded-lipoplexes in the tail vein, the treated mice displayed reduced tumor size as compared to negative controls (LP-miR-NC) and untreated mice. MiR-29b expression in tumor tissue of treated mice was 5-fold higher, confirming the efficient release of miR-29b from DOTMA lipoplexes. As for the biological impact of restoring miR-29b expression, Wu et al. [82] observed a significant decrease in miR-29b oncogenic targets DNMT3B (DNA (cytosine-5-)-methyltransferase 3 beta), CDK6 (Cell division protein kinase 6) and MCL1 (Induced myeloid leukemia cell differentiation protein) with minor toxicity. Nonliving bacterial nanocells (EDVs or TargomiRs) were used as carriers for miR-16 delivery to 26 NSCLC patients in a phase I clinical study (NCT02369198). The targeting moiety of this bacterially derived delivery system was an anti-EGFR bispecific antibody to target EGFR-expressing cancer cells. Tumor growth was impaired after systemic administration of TargomiRs at low dosages. However, dose-dependent toxicities were reported, i.e., anaphylaxis, inflammation as well as cardiac events. Variable response rates were observed with 5% of the patients showing partial response, 68% showing stable disease and 27% showing progressive disease [83]. Based on these observations, the authors recommended to conduct a new trial combining TargomiRs with chemotherapy or immunotherapy in larger groups of patients. More recently, Exosome-Mimetic Nanosystems were engineered with organ specific proteins such as Integrin α6β4 for the targeted delivery of miR-145 mimics to lung adenocarcinoma cells. In vivo experiments were carried out using intraperitoneal or retro-orbital injection of labeled miR-145-EMNs into nude mice bearing lung tumors. Fluorescence was mainly detected at tumor sites and mild off-target effects were found in the liver and spleen [74].

**Table 1:** Examples of in vitro (cell lines) and in vivo studies (preclinical mouse cancer models) that have been conducted to test miRNA-based therapies in the absence or in the presence of accompanying anticancer drugs.

Cancer Type	miRNAs	Delivery System	MiRNA Loading Strategy	Cell Lines	Delivery Route In Vivo	Results	References
Lung cancer	miR-34a	Neutral Lipid Reagent (RNA-LANCEr II)	Encapsulation in phospholipid-oil emulsion	A549, BJ, NCI-H460, Calu-3, NCI-H596, NCI-H1650, HCC2935, SW-900, NCI-H226, NCI-H522, NCI-H1299, Wi-38 and TE353.sk	it, iv	Reduced cell proliferation and colony formation; Tumor growth inhibition	[77]
	miR-34 let-7	Neutral Lipid Nanoemulsions	Encapsulation in phospholipid-oil emulsion	KRAS/TP53-mutated NSCLC cell lines: H358, H23, and H441	iv	Decreased MET and MYC expression; 40% better survival rate	[78]
	miR-29	Cationic DOTMA Lipoplexes	Electrostatic interaction	A549	iv	Decreased expression of miR-29b oncogenic targets DNMT3B, CDK6 and MCL1	[82]
	miR-16	Bacterial Minicells (with EGFR antibody coating)	Loading via non-specific Porin channels	-	iv	Inhibition of tumor growth but dose-dependent toxicities	[83]
	miR-145	Liposomal Exosome-Mimetic Nanoplatforms (Integrin α6β4 coating)	Encapsulation in aqueous phase	A549	ip, ro	Preferential accumulation at tumor sites	[74]
Liver cancer	miR-122	Lentivirus	Viral vector expression system	Mahlavu SK-HEP-1	sc	Reduced ADAM17 expression; Inhibition of tumor growth, angiogenesis, and intrahepatic metastasis	[84]
		Cationic Liposomes	Encapsulation	Sk-Hep-1	it	50% growth suppression of Sk-Hep-1 xenografts; impairment of angiogenesis; Downregulation of SRF, IGF1R and ADAM10	[85]
	Anti-miR-221	PEI-modified PLGA nanoparticles	Electrostatic interaction	HepG2	sc	Inhibition of tumor growth; Increased circulating miR-221	[86]
	miR-199a/b-3p/anti-miR-10b	PEI-Cyclodextrin-PEG polymeric nanoparticles	Electrostatic interaction	Huh-7 PDX	iv	Inhibition of Huh-7 tumor growth by targeting mTOR, PAK4, RHOC and EMT pathways. Tumor suppression on PDX	[87]
	miR-27a Sorafenib	Anti-GPC3 antibody-targeted lipid nanoparticles	Electrostatic interaction	HepG2	-	Suppression of tumor burden; increased apoptosis	[88]
Breast cancer	miR-125a	Liposomes (with hyaluronic acid coating)	Electrostatic interaction	SKBR3, 21MT-1	-	Reduced HER-2 expression	[89]
	miR-34a Doxorubicin	Hyaluronic acid- chitosan nanoparticles	Electrostatic interaction	MDA-MB-231	iv	Enhanced response to chemotherapy	[90,91]
	Anti-miR-21 Adriamycin	PEI graphene oxide nanocarriers	PEI-mediated electrostatic interaction	MCF-7	-	Increased Adriamycin uptake	[92]
	miR-9 miR-21 miR-145	PEI-modified magnetic nanoparticles	Electrostatic interaction	MCF-7	iv	Effective tumor targeting; Reduced tumor burden	[93]
	miR-34	Silica nanoparticles	Electrostatic interaction with added amine groups	Comma Dβ, SUM159pt	it	Inhibition of tumor growth	[94]
Glioblastoma	miR-100 anti-miR-21	Gold-iron oxide nanoparticles (with T7 peptide-cyclodextrin-chitosan coating)	Electrostatic interaction	U87-MG	in	Diagnosis by MRI tracking of gold nanoparticles; Presensitization to temozolomide	[95]
	Anti-miR-21	Cationic polyamine-co-ester	Electrostatic interaction	U87	ced	Apoptosis of GBM cells; Better survival rates	[96]
	miR-34a	Dendritic polyglycerolamine	Electrostatic interaction	Patient-derived GBM cells	iv	Reduced tumor burden	[97]
Thyroid cancer	Anti-miR-146	Invivofectamine	Electrostatic interaction	Cal62	it	Impaired tumor growth; Restored PTEN expression	[98]
	Anti-miR-21	LNA	Chemical modifications	RTL-5	sc	Inhibition of tumor growth	[99]
	miR-204-5p	Lentivirus	Viral vector expression system	TCP-1 BCPAP	sc	Inhibition of tumor growth	[100]
Adrenocortical cancer	miR-7	Bacterial Minicell particles “EnGeneIc Delivery Vehicles” (EDVs) (with EGFR antibody coating)	Loading via non-specific porin channels	NCI-H295R SW13	iv	Inhibition of tumor growth by targeting CDK1/Raf1/mTOR signaling	[101]
	miR-431 Doxorubicin Mitotane	Lipofectamine	Electrostatic interaction	NCI-H295R	-	Reversed EMT phenotype	[102]
Ovarian cancer	miR-200c Paclitaxel	Lipofectamine	Electrostatic interaction	SCOV3	-	Impaired migration and invasion, enhanced chemosensitivity	[103]
	miR-200a miR-141	Lentivirus	Viral vector expression system	SCOV3	-	Improved sensitivity to paclitaxel	[104]
	miR-7 Paclitaxel	Polymeric Nanoparticles (monomethoxy(poly(ethylene glycol))-poly(d,l-lactide- co-glycolide)-poly(l-lysine)	Electrostatic interaction with the poly(l-lysine) chains in the core	SCOV3	iv	Improved sensitivity to paclitaxel and apoptosis of cancer cells through inhibition of EGFR/ERK pathway	[105]
	miR-15a miR-16 Cisplatin	Liposomes	Electrostatic interaction	A2780 A2780-CP20 OVCAR4	iv	Reduced tumor burden; decreased expression of BMI1 oncogene and EMT markers	[106]
	Anti-miR-21	Mesoporous Silica Nanoparticles (with CGKRK peptide coating)	Calcium silicate trapping procedure	OAW42	iv	Reduced tumor mass	[107]
	Anti-miR-21	Gold Nanoparticles	Surface functionalization with amine or thiol groups	-	-	Disrupted cell colony formation ability	[108]
	miR-155	PEI	Electrostatic interaction	OvCa-associated dendritic cells	ip	Boosted immunity and better survival	[109]
Prostate Cancer	miR-34a	Chitosan Nanoparticles	Electrostatic interaction via the protonated amino groups at low pH	PC3	iv	Inhibited tumor growth and metastasis	[110]
	Anti-miR-221	Mesoporous Silica Nanoparticles	Electrostatic interaction within the pore	PC3	-	Less cancer expansion	[111]
	miR-205 Docetaxel	Iron oxide nanoplatforms	Electrostatic interaction	PC3 C4-2	-	Induced apoptosis and Chemosensitization	[112]
	miR-145	SSPEI with R11 peptide coating	Electrostatic interaction	PC3 LNCAP	iv	Impaired tumor growth Enhanced survival	[113]

It = intratumor; iv = intravenous; ip = intraperitoneal; ro = retroorbital; in = intranasal; ced = convection-enhanced delivery; PDX = Patient-Derived Xenografts.

### 4.2. Liver Cancer

Liver cancer is one of the most common malignancies worldwide and the third leading cause of cancer-associated mortality. It has a poor prognosis due to largely ineffective therapeutic options. Surgical removal or liver transplantation is the only curative treatments for early-stage HCC, the most frequent type of primary liver cancer [114]. Alterations of miRNAs landscape and their potential as therapeutic targets in liver diseases, including liver metabolism dysregulation, liver fibrosis and liver cancer have been the focus of several reviews [115,116,117]. Plasma levels of synthetic miRNA antagonists or miRNA mimics distribute broadly after intravenous administration but later accumulate mostly in the liver and kidney and remain high up to 24 h after injection [118]. On the other hand, NPs biodistribution studies have demonstrated that the majority of injected nanomaterials usually accumulate in the liver before undergoing hepatic clearance [119]. This makes liver cancer a good model for testing miRNA-based therapy approaches as this organ can be easily targeted with different delivery systems. Nevertheless, miRNA delivery through NPs to treat HCC has to take into consideration passive and active mechanisms to avoid or delay liver elimination. MiR-122, a highly abundant, liver-specific miRNA that accounts for approximately 70% of the whole hepatic miRNome in humans, was found to be markedly downregulated in HCC. Restoring miR-122 using a lentiviral expression vector in metastatic liver cancer cell lines inhibited migration and invasion in vitro as well as tumorigenesis, angiogenesis, and metastasis in vivo [84]. It was further demonstrated that miR-122 inhibits hepatocellular carcinoma metastasis by modulating ADAM17 (a disintegrin and metalloprotease 17) [84] and cyclin G1 (CCNG1) [120]. Hsu et al. demonstrated that delivery of miR-122 to HCC cells using cationic lipid nanoparticles consisting of 2-dioleyloxy-*N*,*N*-dimethyl-3-aminopropane (DODMA), egg phosphatidylcholine, cholesterol, and cholesterol-polyethylene glycol (LNP-DP1) dramatically downregulated miR-122 target genes [85]. In vivo, LNP-DP1-encapsulated miR-122 mimic induced HCC xenografts growth suppression without causing systemic toxicity. MiR-26a is expressed at high levels in normal adult liver but is dramatically downregulated in both human and murine liver tumors. MiR-26a replacement using AAV as delivery vector potently suppressed cancer cell proliferation and activated tumor apoptosis in vivo, leading to marked suppression of tumor growth [45]. It was further shown that miR-26a arrested the cell cycle at G1 phase in human liver cancer cells by downregulating cyclins D2 and E2. MiR-21 is highly over-expressed in HCC [121]. Inhibition of miR-21 in cultured HCC cells increased expression of PTEN tumor suppressor, and decreased tumor cell proliferation, migration, and invasion [122]. Meng et al. investigated poly(lactic-co-glycolic) acid (PLGA)-based nanoparticle for the delivery of anti-miR-221 to HCC cells and tested its therapeutic efficacy in vitro and in vivo [86]. PLGA nanoparticles encapsulating anti-miR-221 suppressed HCC cell growth, colony formation ability, migration, invasion, and impaired tumor growth in mice. Interestingly, Shao et al. developed a combination therapy by encapsulating miR-199a/b-3p mimics and anti-miR-10b into a polymer-based nanoplatform PEI-βCD@Ad-CDM-PEG (PCACP) to treat HCC. PCACP significantly inhibited HCC cell proliferation and tumor growth by targeting mTOR (mechanistic target of rapamycin), PAK4 (p21-Activated kinase 4), RHOC (Rho-related GTP-binding protein) and epithelial mesenchymal transition (EMT) pathways both in vitro and in vivo [87]. In an elegant study, Sorafenib (SRF), and anti-miR-27a-loaded anti-GPC3 antibody targeted cationic LNPs were developed to treat HepG2 cell xenograft-bearing mice [88]. Combination of SRF and anti-miR-27a (G-S27LN) decreased cell viability and potentiated cell apoptosis compared to SRF alone, suggesting a synergistic anticancer effect. A significant reduction of tumor burden and marked TUNEL positive apoptosis were observed in animals treated with G-S27LN formulation.

### 4.3. Breast Cancer

As HER-2 (Human Epidermal Growth Factor Receptor 2) positive breast cancers account for 30% of cases associated with poor prognosis, more attention is being brought to efficiently target this overexpressed receptor. In this context, in vivo studies in mice models of breast cancer have demonstrated that lentiviral delivery of the tumor suppressor miR-125a-5p reduced tumor growth, metastasis, and angiogenesis by directly targeting HDAC4 (Histone deacetylase 4) [123]. Hayward et al. further showed that transfection of miR-125a-5p in hyaluronic acid-coated liposomes indeed knocked down the HER-2 proto-oncogene in 21MT-1 breast cancer cells. This resulted in reduced migratory and proliferative potential due to inactivation of MAPK and PI3K/AKT signaling [89]. Taking into consideration the overexpressed hyaluronic acid (HA) receptors in breast cancer, HA/miRNA nanoparticles hold great promises for targeted clinical approaches. Interestingly, HA-chitosan nanoparticles were used to co-encapsulate doxorubicin and miR-34a. Deng et al. showed that administration of these formulations into nude mice enhanced the response to chemotherapy and decreased cancer cell migration due to inactivation of Notch signaling by miR-34a [90,91]. In a similar approach, Adriamycin uptake by MCF-7 cells was increased when delivered together with anti-miR-21 in PEI graphene oxide carriers [92]. As cancer cells consistently display alterations in multiple microRNAs, combinatorial strategies have been implemented. Indeed, in vivo studies conducted by Yu et al. showed a 58%-reduction in tumor volume when packaging miR-9, miR-21 and miR-145 sponges into PEI-modified magnetic particles [93]. Recently, Panebianco et al. demonstrated that silica nanoparticles (SiO2NPs) allowed delivery of miR-34a into mammospheres and mammary tumors [94]. MiR-34a/SiO_2_NPs complexes decreased sphere formation efficiency and reduced tumor growth in mice. The levels of well-known target genes of miR-34a such as NOTCH1 (Notch Receptor 1), Cyclin E2 and c-Myc were significantly reduced, indicating the biological activity of delivered miR-34a.

### 4.4. Glioblastoma

Despite conventional therapeutic options involving surgery, radiology, and chemotherapy (mainly Temozolomide), glioblastoma (GBM) remains a lethal malignancy with unmet clinical needs. The implementation of the RNA interference technology provided new insights for GBM gene therapy. For example, miR-21 has been recognized as a major oncomiR upregulated in GBM. It contributes to tumorigenesis by directly targeting PTEN, thus blocking expression of key apoptosis-enabling genes such as caspases and p53. MiR-21 overexpression is also associated with drug resistance, hence chemotherapy failure [124,125]. Conversely, the tumor suppressor miR-100 was shown to trigger the p53 network through regulation of the PLK1 (Polo-Like Kinase gene 1) signaling in tumor-initiating cells [126]. Of note, this apoptotic pathway is also activated by the gold-standard GBM treatment, Temozolomide, suggesting a potential chemo-sensitization via miRNA remodeling [127]. In a combined theranostic-chemotherapeutic approach, gold-iron oxide NPs were used to co-deliver miR-100 and anti-miR-21 into GBM xenograft-bearing mice. The carriers were tailored with a GBM cell-targeting T7 peptide and a cyclodextrin-chitosan polymer layer for specific brain targeting [95]. In vivo experiments were carried out by intranasal inhalation of these nanoformulations to bypass the blood–brain barrier. In parallel, a group of mice received systemic doses of temozolomide. Remarkably, mice co-treated with miR-loaded-NPs and temozolomide chemotherapy showed better survival than animals receiving either miR-NPs or chemotherapy alone, or no therapy. Furthermore, given their magnetic resonance property, it was possible to track gold-iron formulations by MRI imaging. Similar results were obtained by intratumor administration of miR-21-inhibiting NPs named PACE (cationic polyamine-co-ester) [96]. As for most solid cancers, miR-34a was also investigated in GBM for its apoptosis-inducing capacities. When complexed in a dendritic polyglycerolamine (dPG-NH2) cationic carrier, miR-34a stability was enhanced, thus disabling in vitro proliferation and migration of glioma cell lines via C-MET, CDK6, NOTCH1, and BCL-2 inhibition [97]. In vivo studies revealed reduced tumor burdens upon tail vein injection of dPG-NH2-miR-34a polyplexes. Interestingly, the protected miR-34a was able to cross the blood brain barrier with no reported toxicity.

### 4.5. Endocrine Cancers

With regard to endocrine tissues, miRNAs are indeed relevant players given their hormone-like effects with endocrine, autocrine or paracrine regulatory functions mediating intercellular communication [128]. A reciprocal interplay between hormones and microRNAs has been described: miRNAs can alter hormone metabolism via their binding to genes coding for hormones, hormone antagonists, enzymes of hormone biosynthesis, or even hormone receptors [129]. Conversely, many hormones were shown to modulate microRNA expression patterns through regulation of miRNA transcription or biogenesis. Understanding these regulatory feedback loops and how they are perturbed in cancer are critical for the development of miR-based therapeutics and biomarkers in endocrine tissues.

#### 4.5.1. Thyroid Cancer

Thyroid carcinoma is the most common form of endocrine cancers. According to the Surveillance, Epidemiology, and End Results (SEER) registry, thyroid cancers account for 3% of new cancer cases in the US and 0.4% of all cancer deaths with a 2.9-times higher rate in women. Such tumors normally disrupt hormone secretion and are associated with hormone-related complications. Papillary thyroid cancer (PTC) is the most common form of differentiated thyroid neoplasia, which arise from the follicular cells of the thyroid gland. MiRNA profiling in thyroid tumors led to the identification of specific signatures that could be useful for diagnosis and possibly for therapy [130,131]. Among others, miR-146b is highly expressed in PTC and is correlated with pejorative outcome. It was further demonstrated that the tumor suppressor PTEN holds a miR-146 binding site in its 3′-untranslated region. MiR-146b-mediated downregulation of PTEN triggers the PI3K/AKT signaling pathway thus promoting cell proliferation, survival, migration, and invasion. In agreement with these findings, intratumor delivery of miR-146b inhibitors using lipid formulations—namely invivofectamine—suppressed miR-146b-induced aggressiveness in xenograft models [98]. RAS activating mutations have been widely reported in thyroid cancer. Besides their major effects on the global transcription of protein-coding genes, activated RAS proteins have been found to promote the increase of a subset of miRNAs, of which miR-21. Frezzetti et al. showed that LNA-mediated knockdown of miR-21 in RAS-transformed FRTL-5 thyroid cells was able to inhibit markedly the growth of tumor xenografts [99]. The Wnt/β catenin pathway was shown to be activated in PTC as an indirect target of the oncogenic miR-155 [132]. Moreover, miR-155 overexpression in PTC was associated with enhanced survival and colony formation in PTC cell lines. These observations were confirmed in nude mice inoculated with miR-155-transduced TPC-1 cells where larger and highly proliferating tumors were obtained, thus suggesting that silencing miR-155 may be a potential therapeutic strategy for treating PTC. MiRNA replacement therapy has been also conducted in PTC preclinical models. Functional assays by Lianyong et al. showed that miR-204-5p impairs tumor growth through repression of IGFBP-5 (Insuline-like growth factor-binding protein 5). In nude mice, subcutaneous engraftment of human PTC cells stably expressing miR-204-5p induced smaller tumors as compared to controls [100]. In addition to their role in the regulation of cancer hallmarks, miRNAs could also modulate response to adjuvant therapy in thyroid cancer. Approximately 30% of patients with advanced stages of differentiated thyroid cancer are refractory to radioiodine therapy, due to reduced expression of the Sodium Iodide Symporter (NIS) [133]. High levels of miR-146b were shown to disrupt thyroid differentiation and iodide uptake by direct repression of the transcription factor PAX8 and its target gene NIS. Other genes involved in iodide transport mechanisms such as Dehalogenase 1 and Deiodinase 2 are also regulated by miR-146b thus confirming its pivotal role in radioiodine therapy [134]. The authors suggested that miR-146b-3p/PAX8 (Paired box gene 8)/NIS regulatory axis might be a relevant therapeutic target to modulate thyroid cell differentiation and iodide uptake for improved treatment of advanced thyroid cancer. All these important findings are waiting for further exploitation using nanoparticle-based delivery of therapeutic miRNAs in thyroid cancer.

#### 4.5.2. Adrenocortical Cancer

**Figure 3:**
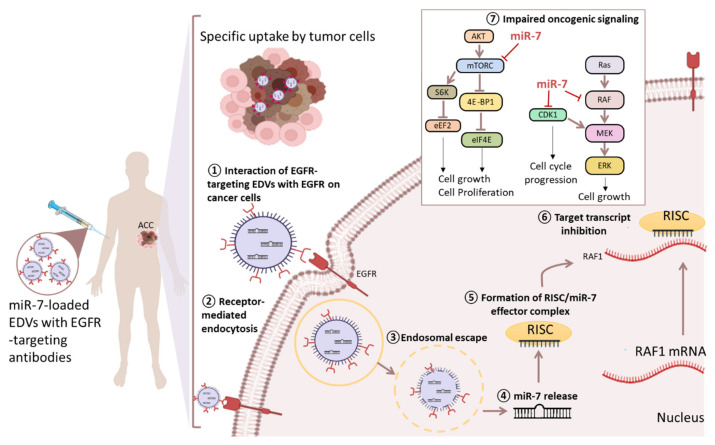
A proposed model in which miR-7 replacement via EDVs (EnGeneIC Delivery Vehicle) in Adrenocortical Carcinoma could inhibit multiple oncogenic pathways including mTOR, MAPK and CDK1 signaling pathways [70]. mTORC: mammalian target of rapamycin Complex; 4EBP1: eukaryotic translation initiation factor 4E- (eIF4E-) binding protein 1; eIF4E: eukaryotic translation initiation factor 4E; S6K: ribosomal protein S6 kinase; eEF2: eukaryotic elongation factor 2; CDK1: cyclin dependent kinase 1.

Adrenocortical carcinoma (ACC) is a rare and highly aggressive malignancy (incidence of 4–12 cases per million per year) which develops in the cortex of the adrenal gland. Cortisol hypersecretion, causing rapidly progressive Cushing’s syndrome, is the most common hormone excess in ACC patients. The clinical outcome of ACC is poor, with a 5-year survival ranging from 15 to 35% [135]. About half of the patients presents with advanced disease or develop local recurrence and distant metastasis after surgery. Complete surgical removal of the tumor remains the mainstay treatment option for ACC. Mitotane, the only FDA-approved drug for this cancer, displays some single-agent activity (10–30% tumor response rates) based on its adrenolytic activity, but its broad clinical use is challenged by an unfavorable toxicity profile [136]. Response to combination of mitotane and cisplatin-based chemotherapies do not exceed 20% for patients with advanced ACC, either recurring or metastasizing [137]. Targeted therapies including inhibitors of IGF (Insuline-like Growth Factor)/mTOR pathway, VEGFRs and other tyrosine kinase receptors such as EGFR and FGFR have been largely ineffective as monotherapy. The multiple genomic and molecular alterations reported in ACC (TP53, Wnt/ß-catenin, and IGFR pathways) include extensive deregulations of miRNA expression [138,139,140,141,142,143,144,145]. Among the most frequently deregulated miRNAs in ACC, miR-483, miR-139-5p, miR-503, and miR-210 were found to be upregulated, whereas miR-195 and miR-335 were found to be downregulated [146]. However, miRNA-based therapeutic approaches for ACC are still scarce as most studies focused on the biomarker potential of tumor or circulating miRNAs [146]. A first preclinical approach was performed using the genetically modified bacterial nanocells (EDVs) to deliver systemically the tumor suppressor miR-7 into a human ACC mouse model [101]. Specific tumor homing was ensured by using EGFR-tailored EDVs. MiR-7-loaded nanoparticles could effectively reduce ACC xenograft growth arising from both an ACC cell line and patient-derived xenografts, without any evidence of off-target effects. Mechanistically, this phenotype was mediated by repression of RAF1, mTOR, and CDK1 (Figure 3). As miR-7 replacement therapy acted synergistically with Erlotinib therapy in head and neck cancer [147], it is crucial to assess whether combination of miR-7 and mitotane would have similar effects in ACC. Such was the case of miR-431, which efficiently sensitized ACC cell lines to mitotane and doxorubicin. In fact, miR-431 was 100-fold underexpressed in patients who were resistant to adjuvant therapy, when compared to sensitive ones. Following transfection of the ACC cell line H295R with miR-431 mimics followed by treatment with doxorubicin or mitotane, H295R cells showed reduced proliferation and increased apoptosis. Restoring miR-431 expression could reverse the EMT phenotype as shown by ZEB1 (Zinc finger E-box-binding homeobox 1) transcription factor repression [102]. These findings support a great potential of miRNA therapeutics for ACC alongside clinical trials based on combined chemotherapy [137]. Jung et al. proposed an experimental setup with liposome-encapsulated chemotherapeutics (L-EDP-M etoposide (E), doxorubicin (D), cisplatin (P), and mitotane (M)) in order to minimize unintended targeting [148]. Treatment of the ACC cell line SW-13-derived xenografts in mice induced necrosis and reduction in tumor size. Interestingly, the research group reported an increased expression of circulating miR-210 in the L-EDP-M-responsive animals. Since miR-210 is a frequently described as an oncomiR in ACC, its release from the tumor to the circulation may be valuable for monitoring response to therapy.

#### 4.5.3. Ovarian Cancer

Intensive analysis of the Cancer Genome Atlas (TCGA) data revealed a large panel of miRNA deregulations in the three ovarian cancer (OvCa) subtypes (serous, endometrioid, and clear cell carcinoma). The miR-200 family proved to be of great prognostic value as it gathers five tumor suppressor miRs arising from 2 genomic clusters, namely miR-200b, miR-200a, miR-429, miR-200c, and miR-141 [149]. MiR-200c upregulation is predictive of good prognosis in OvCa. Its overexpression impaired migratory and invasive capacities of SCOV3 cell lines and significantly increased their susceptibility to microtubule-targeting chemotherapeutics, i.e., paclitaxel (PTX) [103]. Animal studies conducted by Mateescu et al. demonstrated that restoring miR-200a and miR-141 favored tumor growth, but simultaneously enhanced chemosensitivity to PTX, which is among the first-line chemotherapy agents used for OvCa [104]. According to the authors, these miRNAs drive a persistent oxidative stress response through inhibition of p38α, which is associated with an improved sensitivity to PTX. However, a major drawback of this compound is induction of survival, proliferation, and drug resistance upon activation of the EGFR/ERK pathway. Interestingly, miR-7 was shown to suppress this signaling network, thereby suggesting that combining miR-7 therapy with PTX in nanoparticles could enhance sensitivity to chemotherapy [105]. Indeed, upon intravenous administration, PTX/miR-7 nanoparticles revealed anti-cancer properties in OvCa models through the inhibition of PTX-induced EGFR/ERK pathway. Responsiveness to cisplatin chemotherapy was also improved when liposomal miR-15a and miR-16 were injected in a chemo-resistant orthotopic OvCa mouse model [106]. A reduced tumor burden along with decreased expressions of the BMI1 oncogene, the EMT markers as well as the cisplatin transporter ATP7B were reported in treated mice as compared to negative controls. Peptide modified-porous silicon nanoparticles were used by Bertucci et al. [107] to encapsulate an anti-miR-21 LNA. A tumor specific-peptide CGKRK was engrafted on the surface of the nanocarriers for targeted distribution. Internalization, silencing efficiency, and antitumor activity were firstly determined in cultures of OAW42 OvCa cell lines. In mice bearing subcutaneous xenografts, five doses of anti-miR-21 formulations at 25 mg/kg injected in the tail vein every other day significantly reduced the tumor mass [107]. Another study demonstrated that gold nanoparticles were attractive platforms for anti-miR-21 delivery to OvCa cells as they efficiently silenced endogenous miR-21 and disrupted cell colony formation [108]. Lactic-co-glycolic acid-modified polyethylenimine (LGA-PEI) could successfully transfer miR-520h mimics into ovarian xenograft tumors [150]. Along the same line, miR-155 loaded-PEI nanocomplexes were used for OvCa immunotherapy. While miR-155 was demonstrated to have an immune-stimulatory role [151], it was found underexpressed in OvCa-derived dendritic cells. After intraperitoneal injection, miR-155-PEI were selectively taken up by dendritic cells residing in ovarian tumors, thus boosting anti-cancer immunity and increasing mice survival rates by 65% [109].

#### 4.5.4. Prostate Cancer

The taxanes docetaxel and cabazitaxel remain the standard of care for prostate cancer (PCa). However, drug resistance remains a major issue, which imply the development of new therapeutic strategies. There has been rapidly growing interest in alternative therapeutic molecules such as miRNAs for PCa. For example, systemic injection of miR-34a-enriched-chitosan nanoparticles inhibited prostate tumor growth in subcutaneous xenograft models and prevented bone metastasis [110]. Besides downregulation of its target genes including MET, Axl, and c-Myc, nanoparticle-mediated restoration of miR-34a expression in PC3 cells induced apoptosis and autophagy, and decreased PC3 cell proliferation, invasion, and migration [110]. MiR-221 is one of the most studied oncogenic miRNA in prostate cancer. By inducing p27 cell cycle checkpoint arrest, miR-221 supports uncontrolled proliferation, hence cancer expansion. Moreover, high circulating levels of miR-221 were detected in patients with PCa. This has led Farina et al. to propose a therapeutic approach consisting of miR-221 inhibitor encapsulation into mesoporous silica nanoparticles (MSN) [111]. MSN are biocompatible nanoparticles with a high molecular loading capacity and a possible controlled release of payload. MiR-221 mimic-loaded MSN were successfully delivered to PC3 cell lines where they recapitulated the biological effects of miR-221 [111]. The anti-tumor function of miR-205 was highlighted in prostate cancer using magnetic iron oxide core nanoparticles coated with PEG-PEI layers [112]. MiR-205 nanoplatforms were internalized into PC3 and C4-2 cells as measured by flow cytometry analysis. Western blot analysis revealed an important induction of pro-apoptotic proteins such as cleaved PARP and cleaved Caspase 3 after treatment with miR-205-nanoparticles and Docetaxel. Globally, these formulations reversed cancer hallmarks with a marked anti-migratory and anti-invasive effects as well as chemo-sensitization in vitro [112]. An original approach based on the combination of chemically modified PEI (disulfide linkage in the branched PEI or SSPEI) with the cell permeable peptide R11 (R11-SSPEI nanocarriers) was set up by Zhang et al. [113]. Taking advantage of R11 specific uptake by prostate cancer cells in vivo, they demonstrated that R11-SSPEI nanomaterials were able to deliver miR-145 in intraperitoneal prostate cancer models [113]. Northern blotting of tumor tissue after three weeks of treatment revealed a substantial increase of miR-145 levels in the treated group, thus underscoring the excellent transfection efficiency of PEI formulations. Importantly, MiR-145 overexpression significantly impaired tumor growth and prolonged mice survival.

## 5. Challenges in the Clinical Translation of miRNA Therapeutics

More than fifty therapeutic siRNA programs have entered clinical trials in the past decade (phase I, II, and III) [152]. Patisiran and givosiran (Alnylam Pharmaceuticals), two siRNA-based drugs, were approved by the Food and Drug Administration in 2018 and 2019 for hereditary transthyretin-mediated amyloidosis and acute hepatic porphyria, respectively [153,154]. About fifteen phase I-, II-, and III-programs based on siRNA drugs are dedicated to the treatment of diverse cancer types [152]. Despite such successes in clinical development, several clinical trials have been discontinued, indicating that there are still several challenges to overcome before the clinical application of RNAi-based therapies becomes widespread. These challenges are even more significant for miRNA-based therapies.

So far, only 10 miRNA-based drugs have reached clinical trials with none of them entering Phase III and half of them were halted. MiRNA-based therapy programs for cancer treatment are mainly driven by four biopharmaceutical companies, including miRagen Therapeutics (Boulder, CO, USA), Regulus therapeutics (San Diego, CA, USA), Mirna Therapeutics Inc. (Carlsbad, CA, USA) and EnGeneIC (Sydney, Australia). MiRagen Therapeutics is performing clinical trials of MRG-106 (Cobomarsen, an inhibitor of miR-155) for the treatment of cutaneous T-cell lymphoma, chronic lymphocytic leukemia, diffuse large B-cell lymphoma, and adult T-cell leukemia/lymphoma (NCT02580552, NCT03713320). EnGeneIC is currently testing TargomiRs as 2nd or 3rd Line Treatment for patients with recurrent malignant pleural mesothelioma and non-small cell lung cancer (NCT02369198). The first miRNA-based drug entering clinical trials was Miravirsen, an antagomiR targeting miR-122, as a therapy against Hepatitis C Virus (HCV) infections (Santaris Pharma, Roche Pharmaceuticals). Miravirsen showed a strong efficacy by reducing viremia in patients with HCV [155,156,157] and underwent multiple phase II clinical trials (NCT01200420, NCT01872936, NCT02031133, NCT02508090). Regulus Therapeutics developed another miR-122 inhibitor, RG-101, an *N*-acetyl-d-galactosamine-conjugated antagomiR which showed considerable efficacy in patients with HCV. However, some serious adverse events of severe hyperbilirubinemia led the FDA to suspend the trial. MRX34, a first-in-class cancer therapy developed by miRNA Therapeutics was delivered as a mimic of miR-34 encapsulated into a liposome-formulated nanoparticle (NOV40). MRX34 displayed a strong activity in melanoma, hepatocellular carcinoma, NSCLC, and renal carcinoma (NCT01829971). Unfortunately, miRNA Therapeutics halted the phase I due to multiple immune-related severe adverse events. These successive failures indicate that miRNA-based therapies are still awaiting their Eureka moment.

Delivery systems and administration routes, dosage concerns and off-target effects remain major challenges to be overcome for the development of miRNA-based therapies for cancer and other diseases. Despite a great number of preclinical studies in mouse models of cancer, only a very small number of miRNA candidates have reached clinical development so far. Performing rigorous pharmacokinetics (absorption, distribution, metabolism, and excretion or ADME) studies in animals will provide a basis for anticipating how miRNA mimics/antimiRs will behave in humans. As detailed earlier, nanotechnologies provided versatile platforms for safe biomolecule delivery (polymers, lipid compounds, inorganic nanomaterial). Nanoparticle-based delivery of miRNA aims to increase therapeutic efficacy, decrease the effective dose, and/or reduce the risk of systemic side effects. However, most of these systems have yet to reach their testing in humans. Hence, the challenge is to establish functional, yet biocompatible carrier systems for miRNA therapy. Indeed, nanoparticles are potent reservoirs in which molecular cargo can be particularly enriched. Due to their synthetic malleability, polymeric biomaterials are tailored for specific applications with surface functionalization, high active payload, and minimized toxicity. MiRNA mimics or inhibitors could be therefore shielded from the injection site to the targeted area. This mechanism mirrors the natural shielding of endogenous miRNAs by extracellular vesicles such as exosomes. However, the applicability of nanocarrier formulations for drug administration depends on several parameters including their average diameter and their polydispersity index. Controlling and validating these parameters are of major importance for nanoparticle circulating time, biodistribution and cellular uptake with a view of their effective clinical applications. Other parameters related to charge, shape, surface chemistry, and clearance are also key determinants for nanoparticle fate. Integration of miRNAs, coatings and targeting agents into a single nanocarrier requires multiple steps in the production process. These structural and physicochemical complexities contribute certainly to the slow rates in clinical translation since they hamper large-scale manufacturing by the pharmaceutical industry. Simplification in the design of nanoparticles should allow efficient and reproducible large-scale manufacturing. The EPR effect of nanoparticles in tumors has long stood as an important driver of cancer nanomedicine. However, the reliability of the EPR effect in human patients have been recently debated as the extent of nanocarriers accumulation varies profoundly between patients and tumor types [158]. The mechanism by which nanoparticles enter solid tumors appears more complex than previously thought and probably involves active trans-endothelial pathways [159]. The EPR-dependent drug delivery is compromised by high tumor interstitial fluid pressure and poor blood flow inside human tumors. Despite nanoparticle stabilization and surface manipulations, perfect tumor targeting is not yet achieved [160] Liver and spleen remain the first accumulation sites for nanoparticles due in part to their fenestrated endothelium. Thus, these organs are major barriers to clinical translation of nanomaterials administered intravenously [161]. Understanding the mechanisms behind this accumulation more extensively will help develop new strategies for tumor targeting and liver or spleen escape.

Dosage concerns must be addressed before introduction of miRNA therapeutics into the clinic because an overdose of miRNA mimics/antimiRs may amplify off-target adverse effects, non-specific immune responses, and toxicities. Dose finding in miRNA therapy studies is complex because exposing patients to either a non-active dose or a potentially toxic dose is not ethical. The initial dose for a phase I/II trial is extrapolated from preclinical animal and cell studies to humans. Several variables should be accounted for, including the differences in size and volume between animal and human organs and the spread of the volume delivered. This further underlines the importance of proper dose-range finding studies in large animal models (such as non-human primates) to fill the gap between preclinical research in mouse cancer models and clinical research in cancer patients. In addition, administration routes of oligonucleotide drugs are still problematic since they are prone to nuclease digestion with a bloodstream half-life of only a few minutes. Currently, miRNA mimics/antimiRs can only be administrated through intravenous or subcutaneous routes. The development for oral delivery vehicles will be a key step in advancing this class of drugs to clinical use in patients. Most commercial miRNA mimics/antimiRs undergo different chemical modifications or length changes to increase their stability, which may introduce variations in their activity, pharmacokinetics, and biodistribution. Thus, it is important to characterize each candidate miRNA drug and to evaluate the impact of its specific modifications in early stage of preclinical evaluation. Information on the half-life of the miRNA mimics/antimiRs and the turnover rate of the miRNA targets is mandatory to determine dosing and dosing schedules. For example, measurement of the rate of clearance of antimiRs would allow to replace only the miRNA molecules that are cleared or those required to sequester newly synthesized miRNAs. Defining the doses required to achieve total endogenous miRNA sequestration with antimiRs or endogenous physiological miRNA concentration with miRNA mimics is a key stage of nonclinical toxicity and pharmacokinetic studies. The selected concentration of miRNA mimics/antimiRs should completely silence or upregulate a limited number of target mRNAs in a cell. Any antimiR given in excess of the dose required to fully sequester the available miRNA target will produce non-target-related effects. For example, earlier work showed that LNA-modified anti-miR-122 oligonucleotides could upregulate miR-122 target aldolase A in non-human primates at much lower dose of 1–25 mg/kg [162], compared to the previously reported dose of 120–240 mg/kg of cholesterol-conjugated oligonucleotides in mice [163]. It is reasonable to expect that solving miRNA-dosing issues will be also facilitated by continuous improvement in miRNA target prediction tools and validations of true miRNA targets. Interestingly, Zhang et al. [152] recently analyzed the reasons for the delayed development of miRNA-based therapies compared to siRNA-based therapies. Combining clinical trial information [164], Drugs@FDA database, target prediction softwares and gene ontology enrichment tools allowed them to conclude that the serious immune-related adverse events that led to the discontinuation of MRX34 were due to a “too many targets for miRNA effect” (TMTME) on several genes involved in cytokine and interleukin signaling in the immune system [152]. A combination of tissue specific knockout mouse models and advanced molecular biology techniques will allow us to determine miRNAs target-selectivity and will help us to define the specific contribution of a single miRNA in controlling a biological pathway and gene network in different tissues. This will have major implications for the design of dosage for clinical trials to minimize ineffective and potentially toxic over exposures.

Another challenge is the current regulatory gap for both nanomedicines and RNAi-driven therapies, including miRNA-based therapies [165]. The lack of clear regulatory and safety guidelines for quality control and safety has delayed the development of these products toward effective clinical translation. The increased number of novel polymeric nanomaterials, complex polymeric-based nanoformulations and chemical modifications require appropriate regulatory rules to help in miRNA drug assessment (good manufacturing practices, production process, and quality controls). Simplification in formulation design could be a key step in the evaluation by regulatory authorities. On the other hand, the cost–benefit ratio is another limitation to the clinical translation of miRNA-based therapies when compared to existing anti-cancer therapies, due to the high cost of both miRNA biology products and emerging nanocarriers, which are more complex in structure and more expensive than conventional drugs [166]. The fact that the healthcare system is different in each country is a threat for pharmaceutical companies who want to invest at the international level. The decrease of financial resources and the lack of socio-economic validation studies may neutralize innovative advances. This means that only developed countries will be able to advance miRNA-based therapy programs in the forthcoming years. Among all the countries, North America is expected to remain at the forefront and hold the highest position in the global miRNA market. In the USA, this is attributable to the increasing miRNA clinical trials launched to develop advanced therapeutic solutions. In Europe, growing government funding for the startups for R&D activities to develop novel miRNA-based therapies might allow the region to hold the second position in the market.

## 6. Conclusions

The pleiotropic action of miRNAs suggests that targeting these molecules could efficiently reverse phenotypes of multifactorial pathologies like cancer. As they are short-sequence molecules naturally produced by the cell, miRNA inhibition or replacement are relatively easy and hold great promises for clinical translation [156,167]. The power of Systems biology will allow a better understanding of the high complexity of miRNA-mediated gene regulatory networks and hence, a better evaluation of the therapeutic value of miRNA drugs. The relevance of miRNAs as anti-cancer agents is supported by 11,439 studies referenced in Pubmed under the search terms ‘’microRNA’’ AND “cancer therapy”. The field of nanotechnology is now mature enough to envisage reproducible scale-up for potential clinical studies in the next few years. Messenger RNA-based anti-Covid-19 vaccines are a groundbreaking innovation in nanomedicine and a huge scientific achievement in a very short period of time that could help some of the most promising miRNA nanocarriers to reach the market [168]. Moreover, regulatory authorities gained awareness of nanoparticle use for drug delivery given that several liposomal drugs are now on the market, directly paving the way for miRNA therapeutics to the clinics.
